# Schistosomiasis and soil-transmitted helminthiasis in Rwanda: an update on their epidemiology and control

**DOI:** 10.1186/s40249-016-0212-z

**Published:** 2017-03-01

**Authors:** Nadine Rujeni, Domenica Morona, Eugene Ruberanziza, Humphrey D. Mazigo

**Affiliations:** 1 Department of Biomedical Laboratory Sciences, School of Health Sciences, College of Medicine and Health Sciences, University of Rwanda, P.O. Box 3286, Nyarugenge, Rwanda; 2 Department of Medical Parasitology, School of Medicine, Catholic University of Health and Allied Sciences, P.O. Box 1464, Mwanza, Tanzania; 3 Rwanda Biomedical Center, Institute of HIV, Disease Prevention and Control, Malaria and Other Parasitic Diseases Division, Neglected Tropical Diseases and Other Parasitic Diseases Unit, Kigali, Rwanda

**Keywords:** Schistosomiasis, Soil-transmitted helminths, Control, Epidemiology, Rwanda

## Abstract

Even though Rwanda lies within a region that has a high prevalence of schistosomiasis and soil-transmitted helminth (STH) infections, epidemiological information regarding these infections in the country remains scarce. The present review attempts to compile the available data on schistosomiasis and STHs, from 1940 to 2014, to provide an insight on the epidemiological profile of these infections. This information will, in turn, support the design and implementation of sustainable control measures. The available records indicate that only *Schistosoma mansoni* and all the major species of STHs are endemic in Rwanda. In 2008, the national prevalence of *S. mansoni* was reported to be 2.7%, ranging from 0 to 69.5%, and that of STH infections was 65.8% (diagnosed using the Kato-Katz technique). The prevalence of these infections varies from one district to another, with schoolchildren remaining a highly affected group. The main control approach is mass drug administration using albendazole and praziquantel, mostly targeting school-aged children in school environments. In 2008, adult individuals living in areas with a prevalence of *S. mansoni* ≥30% were also included in the mass drug administration programme. However, despite Rwanda achieving an almost 100% coverage of this programme in 2008–2010, the transmission of *S. mansoni* and STHs continues to take place, as illustrated by the most recent surveys. If Rwanda is to achieve sustainable control and elimination of schistosomiasis and STHs, there is a need to revise the country’s control strategy and adopt an integrated control approach that involves a combination of measures.

**Electronic supplementary material:**

The online version of this article (doi:10.1186/s40249-016-0212-z) contains supplementary material, which is available to authorized users.

## Multilingual abstracts

Please see Additional file 1 for translations of the abstract into the six official working languages of the United Nations.

## Introduction

Schistosomiasis remains one of the most prevalent neglected tropical diseases (NTDs), causing significant morbidity in Sub-Saharan Africa (SSA) [1–3]. Worldwide, schistosomiasis is estimated to affect over 290 million individuals [3], with more than 779 million individuals living in high-transmission areas [1, 2]. Of the infected individuals, 93% live in SSA [1, 2] and approximately 76% live in high-transmission areas. An estimated 120 million individuals have schistosomiasis-related symptoms and the disease accounts for over 2.8 million years lived with disabilities [3, 4]. School-aged children (SAC) harbour the highest prevalence and intensity of schistosome infections, while a lower intensity of infection is observed in adults [1, 4]. In recent years, it has been reported that pre-schoolchildren (PreSAC) from SSA are infected and carry a high intensity of infection [1, 4]. The lower intensity of infection observed in adults might be explained by behavioural changes towards risk areas such as avoiding water contacts or risk areas that come with an increase in age and slower development of partial immunity [5–8]. However, in high-transmission areas and in areas with high frequency of human water contact associated with economic activities, a large number of both young adults and mature adults are infected with the disease [6, 7].

In SSA, the most important species causing schistosomiasis are *Schistosoma mansoni* and *S. haematobium* [9]. *S. mansoni* causes intestinal schistosomiasis and its chronic form is mainly characterised by bloody diarrhoea, bowel ulceration, hepatomegaly, periportal fibrosis that can lead to portal hypertension, oesophageal varices and hematemesis [4, 9]. In SSA, around 8.5 million cases of chronic hepatosplenic schistosomiasis disease are attributed to the *S. mansoni* infection [4, 10]. *S. haematobium* is the aetiological agent of urogenital schistosomiasis and is primarily characterised by urinary bladder pathology, haematuria, dysuria and hydronephrosis [4, 9]. A previous study indicates that *S. haematobium* is responsible for 10 million cases of hydronephrosis in SSA [4]. Emerging evidence also reveals that urogenital schistosomiasis increases the risk of acquiring and transmitting HIV, especially among women of reproductive age [11].

Similarly, soil-transmitted helminths (STHs) continue to be a serious public health problem worldwide, with more than 1.75 billion people estimated to be infected [3, 12–14]. Of these, about 471 million, 477 million and 804 million are estimated to be infected with hookworms, *Trichuris trichiura* and *Ascaris lumbricoides*, respectively [3]. These infections mainly affect the poorest and most deprived communities, which are characterised by poor sanitation [14]. Soil-transmitted helminths are mainly prevalent in the rural areas of SSA, the Americas, China and East Asia [14]. Pre-school and school-aged children carry the highest prevalence and intensity of STHs, with available data indicating that over 270 million PreSAC and over 600 million SAC live in areas characterised by an intense transmission of these parasites [1]. Another estimate indicates that between one-quarter and one-third of the Sub-Saharan African population is infected or co-infected by one or more species of STHs [14]. The common STH species are hookworms, *A. lumbricoides* and *T. trichiura* [15, 16]*.* Of the three STH species, hookworm infection appears to be widely distributed in SSA, with an estimated 198 million people infected, of which 40–50 million are SAC [1, 14, 17]. Similarly, *A. lumbricoides* and *T. trichiura* are estimated to infect 173 million and 162 million individuals, respectively, in SSA, with 81 million of those infected being SAC [14]. In this group, these infections are associated with growth retardation, anaemia, decreased cognitive function and increased school absenteeism [12].

Several attempts have been made to map the geographical distribution of schistosomiasis and STHs in various countries in SSA (http://www.thiswormyworld.org/) [18–20]. These maps remain important tools for planning, resource allocating, implementing and evaluating control programmes. However, these maps have a limitation in that they cannot clearly predict micro-epidemiological features and micro-distribution of these infections in remote areas of the African continent [19]. For example, these maps may not show the socio-economic factors that may contribute to re-infection after treatment or persistence of infections. Thus, a review of the available web-based and grey literature on the micro-geographical and micro-epidemiological distribution of these infections in various countries, such as Rwanda, may provide supplementary information to guide control programmes at the local level.

Rwanda is a country in which the public health system is still recovering from years of destruction as a result of political problems, thus there is a paucity of information on the geographical distribution and risk factors relating to a number of tropical diseases, including schistosomiasis and STHs [21]. In recent years, there have been national, non-governmental organisation and international community efforts to understand the epidemiology of these infections. However, epidemiological information generated by past and present surveys is not easily accessible via the public domain, and where information is available it has not been updated and therefore cannot be readily used for planning and implementing sustainable control programmes [21].

In the attempt to fill this gap, the present paper reviews the available literature on the epidemiology and transmission of schistosomiasis and STHs in Rwanda, and highlights the strengths and weaknesses of control efforts. The main objective is to provide an insight into the epidemiological profile of these infections in Rwanda based on past and recent surveys. This information is important for understanding the existing situation in relation to these infections, tracking the trends across the different regions of the country, and understanding the effect of control programmes on the prevalence and intensity of infections. In addition, this information provides a framework for evaluating the effects of the implemented control approaches and the cost-effectiveness of interventions.

## Method for literature search

The PubMed, Global Health and Google Scholar databases were searched for original articles exploring the epidemiology and control of schistosomiasis and STHs in Rwanda.

The following combinations were used as keywords to search for literature on schistosomiasis: ‘helminths’ and a combination of the following words in permutations – ‘epidemiology + control + Rwanda’ , ‘epidemiology + mass drug administration + Rwanda’ , ‘*Schistosoma mansoni* + Rwanda’ , ‘*Schistosoma haematobium* + Rwanda’ and ‘*Biomphalaria*/*Bulinus* + Rwanda’.

Similarly, for STHs, the following combinations were used: ‘epidemiology + *Ascaris*’ , ‘epidemiology + *Trichuris* + Rwanda’ , ‘epidemiology + hookworm + Rwanda’ and ‘epidemiology + *Ascaris* + control’.

Through this search, 10 publications on the prevalence and intensity of infections and the impact of mass drug administration (MDA) were identified. An additional search of the references identified two more papers. The search specified only publications that were written in the English language and covered a period from 1940 to 2014.

## Epidemiology of schistosomiasis in Rwanda

### Geographical distribution, prevalence and intensity of infection

Historical epidemiological surveys have reported on the transmission of schistosomiasis in Rwanda since the late 1940s [22]. Reports from 1947 indicate that Lakes Bulera and Ruhondo, in Northern Rwanda, were the main sources of infection [22]. In the mid-1970s and early 1980s, the transmission of intestinal schistosomiasis was reported in many areas of the country, with the majority of those infected being children aged 5–10 years [22].

Epidemiological surveys carried out by the Rwandan Ministry of Health at the time noted that Lakes Ruhondo, Bulera, Kivu, Muhazi, Rweru, Mugesera and the swampy areas of Nyagatare district were potential areas for the transmission of *S. mansoni* in the country (see Table 1) [21, 23–26]. The lake basins and swampy areas are potential breeding sites of the snail intermediate hosts, which contribute to the parasite’s life cycle [23]. Indeed, early malacological studies identified the freshwater snails *Biomphalaria* spp. as the intermediate hosts involved in the transmission of *S. mansoni* in Rwanda [23]. This is consistent with findings of 2012 from the Eastern Province of the country, which revealed a high number of *Biomphalaria* spp. infected with *Schistosoma* cercariae [23]. There is a lack of information on the specific species of *Biomphalaria* snails involved in the transmission of *S. mansoni* in Rwanda, though one report indicated *B. pfeifferi* as the main species transmitting the parasite around Lake Ruhondo. The presence of *Bulinus* spp. (freshwater snails that are intermediate hosts for *S. haematobium*) were noted in 2012, but none of the snails observed were infected with cercariae [23].

**Table 1:** Epidemiological studies reporting on the prevalence and intensity of infection of schistosomiasis and STHs in Rwanda

Authors	Parasites	Major findings	Geographical area
[23, 25] 2008	Soil-transmitted helminths-hookworm, *T.trichiura* and *A.lumbricoides* - *S. mansoni* and *S.haematobium*	A total of 8 313 randomly sampled school children from 136 schools in 30 districts in Rwanda. Single stool sample collected from each school child and processed using Kato-Katz method and urine filtration test for detection of *S.mansoni* and STH. Dipsticks and urine filtration test were used for diagnosis of *S. haematobium*. Six species of intestinal helminths were identified with an overall STH infection prevalence of 65.8% with *A. lumbricoides* (38.6%) and hookworms (31.6%) most common. Co-infection of *A. lumbricoides* and *Trichuris trichiura* was found to be particularly common	All geographical areas of Rwanda
	*S. mansoni* and *S.haematobium*	*-S.mansoni* was common along the large water bodies, overall national prevalence was 2.7%, range from 0 to 69.5%. - No cases of *S.haematobium* was detected.	
[24] 1981	*S.mansoni*	A total of 4 751 individuals aged 0- > 30 years were examined single feacal smear on single stool sample. Of these 107 (2.17%) from north and 69 (3.47%) had *S.mansoni* infection.	Bulera south and north
		A total of 5 739 individuals aged 0- > 30 years were examined using single feacal smear on single stool sample. Of these 5.9% (135) from north and 236 (6.8%) had *S. mansoni* infection. Note: No parasitological technique mentioned.	Ruhondo south and north
[26] 2008	*S.mansoni* STH-hookworm, *A.lumbricoides*, *T.trichiura*	A total of 1 605 individuals, 1485 children aged 5-17 and 120 adolescent aged 18–20 years were screening using Kato Katz techniques on duplicated thick smears prepared from single stool sample. Of all the individuals, 0.25, 0.75, 2.8 and 6.04% were infected with *S.mansoni*, hookworm, *T.trichiura* and *A.lumbricoides*. Almost 21.2% were co-infected with *A.lumbricoides* and *T.trichiura* parasites.	Lake Bulera and Ruhondo, Musanze district
[27] 2010	*S.mansoni*	A total of 270 individuals (children and adults) were screened using duplicated Kato Katz thick smears technique on single stool sample. Of these participants, 20.1% were infected with variation of prevalence between villages ranging from 1.6 to 30.1%.	Lake Rweru, Bugesera district
	STH	The prevalence of hookworm was 33%, 22% for *T.trichiura* and 12.2% for *A.lumbricoides*.	
[29] 2015	*S. mansoni*	A total of 311 school children from four primary school were examined for presence of *S.mansoni* eggs using Kato Katz technique on single stool sample. Overall, 62.1% of the children were infected with S.mansoni infection. The overall feacal egg count (eggs per gram of faeces) was 176.9 epg.	Nkombo island, Lake Kivu, Rusizi district
[37] 2014	STH	A total of 662 school children (301 from rural and 321 from urban) with median age of 10.2 years. Stool sample were examined using Polymerase Chain Reaction (PCR) and the overall prevalence of STH was 13% in urban and 38% in rural areas. *A.lumbricoides* accounted for 95.6% of all detected helminths.	Butare and Huye district, southern province

There is limited information on the prevalence and intensity of the *S. mansoni* infection in Rwanda. Past epidemiological surveys in 1981 reported a prevalence of 6.5% along Lake Ruhondo in Northern Rwanda [22]. The results were based on a single faecal smear observation [22]. It should be mentioned that this study was criticised for not describing the laboratory technique used to screen for *S. mansoni* infection [22].

In 2007, the Schistosomiasis Control Initiative (SCI), in collaboration with Columbia University in the United States, Geneva Global and the Global Network for NTDs, with funding from the Legatum Group, initiated an integrated NTD programme in Rwanda through the Access Project, which aimed at mapping the distribution of NTDs [21, 27]. A nationwide epidemiological survey, which included 8 313 schoolchildren (aged 10–16 years) from 136 schools in 30 districts, revealed that *S. mansoni* was endemic in 18 districts, with prevalence rates ranging from 10 to 50% in eight districts and over 50% in two districts [21, 27, 28]. The overall national prevalence of *S. mansoni* based on the Kato-Katz diagnostic technique was estimated to be 2.7%, with variations between school/districts ranging from 0 to 69.5% [21, 27]. In this survey, although few cases of microhaematuria were detected, no cases of *S. haematobium* infection were confirmed by the urine filtration technique [21, 27].

Epidemiological surveys in 2010 and 2015 conducted in areas not previously included in the nationwide epidemiological surveys have exposed a higher prevalence of *S. mansoni* infection in areas around Lake Rweru and Nkombo Island [24, 25]. Along Lake Rweru, an epidemiological survey (based on the Kato-Katz diagnostic technique) conducted among 270 SAC and adults revealed a prevalence of 20.1%, with variations between the study villages (ranging from 1.6% in Mujwiri to 30.1% in Mazane Island) [25]. The age-based classification of the prevalence indicated that the age group 5–16 had the highest prevalence of the infection [25].

The initial national mapping survey in 2008 classified the Rusizi district as an area with a low endemicity of *S. mansoni* infection, however, a recent survey reported a prevalence of 62.1% (based on the Kato-Katz diagnostic technique) among 311 SAC aged 10–19 years on Nkombo Island located in this district [24]. A variation in prevalence between schools, ranging from 28.6 to 77.9%, was reported [24].

A recent systematic review and geostatistical analysis reported an overall prevalence of 3.8% for *S. mansoni* based on data that were available in 2012. The review further indicated that an estimated 2.9 million, 299 000 and 30 000 individuals were living in areas characterised by low, moderate and high risk for schistosomiasis transmission [29].

**Fig. 1:**
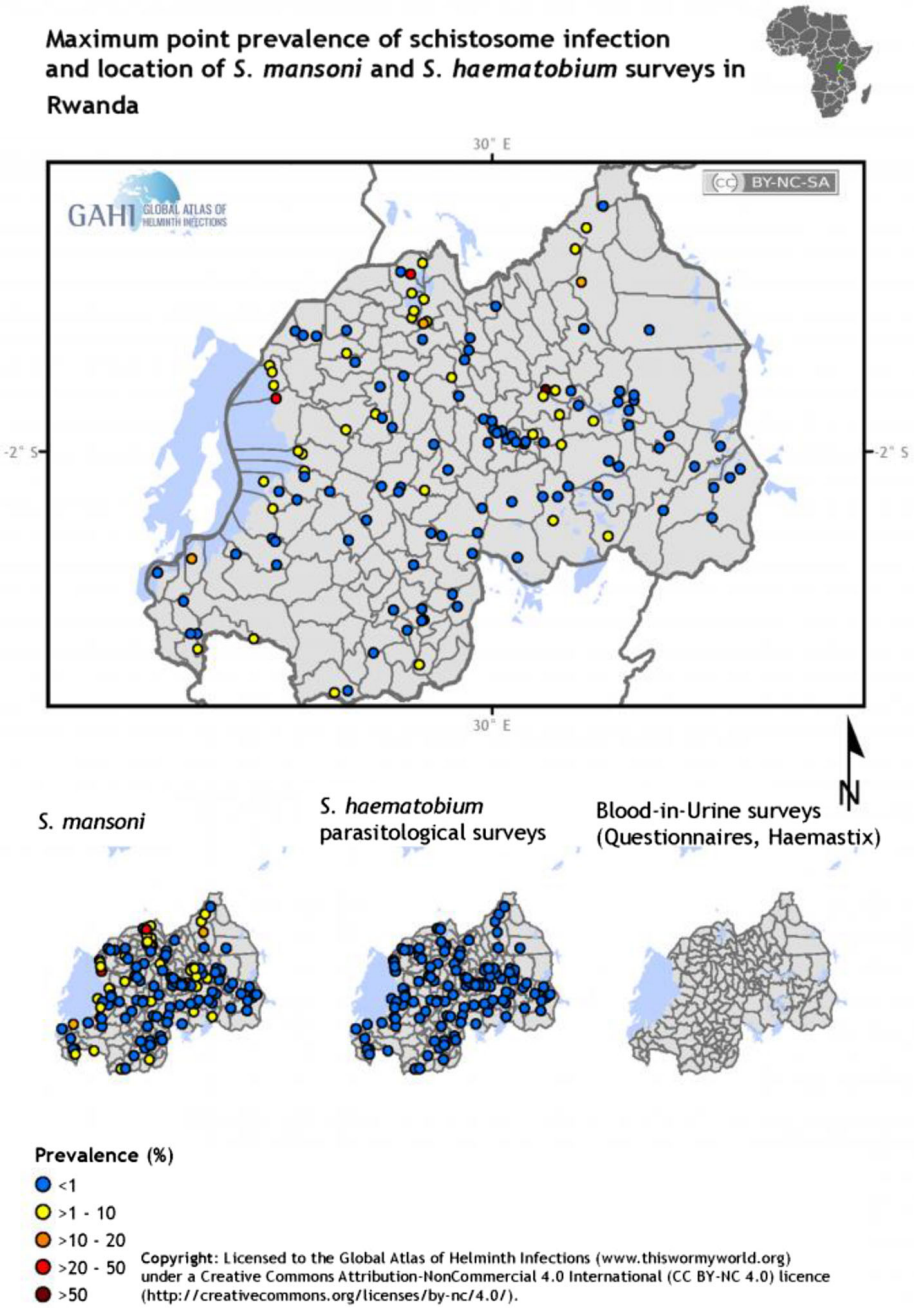
Distribution of schistosomiasis in Rwanda. Map shows maximum point prevalence of schistosome infections and locations of *S. mansoni* and *S. haematobium* surveys in Rwanda. (http://www.thiswormyworld.org/maps/2013/distribution-of-schistosomiasis-survey-data-in-rwanda)

In general, these findings clearly indicate that *S. mansoni* is focally transmitted in Rwanda and that prevalence of infection varies substantially from one epidemiological setting to another. The variations in prevalence may partly be explained by variations in the geographical distribution of potential risk factors, such as large water bodies, and differences in socio-economic status among the communities living in areas with different transmission intensities. In addition, the use of the Kato-Katz technique, which is a less sensitive method when used to diagnose infection among a lightly infected population, may explain the observed variation in prevalence. Thus, the use of more sensitive techniques for mapping, such as the circulating cathodic antigen, is highly recommended [30]. Figure 1 shows the distribution pattern of *S. mansoni* and *S. haematobium* in Rwanda.

In relation to the intensity of infection, most surveys have indicated that the majority of *S. mansoni*-infected individuals carry a light intensity of infection and only a few are moderately or heavily infected [21, 24, 25, 27]. However, in areas with intense transmission such as Nkombo Island and along the shores of large water bodies such as Lakes Rweru and Muhazi, *S. mansoni*-infected individuals are moderately or heavily infected [24, 25]. These findings corroborate the findings reported elsewhere about *S. mansoni*-endemic areas in SSA [31–33].

## Geographical distribution, prevalence and intensity of infection of soil-transmitted helminths in Rwanda

Soil-transmitted helminths, which include *A. lumbricoides, A. duodenale* (hookworm) and *T. trichiura*, are among the common neglected tropical parasitic infections that occur in Rwanda. These infections are known to affect PreSAC and SAC [34, 35]. In 2007/8, the geographical distribution of STH infections in Rwanda was reviewed through a countrywide mapping among schoolchildren [21, 27]. A total of 8 313 SAC from 136 primary schools in 30 districts were included in the screening programme [21, 27]. The overall prevalence of any STH was 65.8%, with a prevalence of 38.6% for *A. lumbricoides*, 31.6% for *A. duodenale* and 27% for *T. trichiura* [21, 27].

National surveys have shown a wide distribution of STH infections in Rwanda with significant variations between districts [21, 27]. For instance, in Huye district (Southern Province), in 2008, the prevalence of *A. lumbricoides* was 61%, of hookworm it was 31% and of *T. trichiura* it was 13% (see Table 1) [21]. There was also a variation in the prevalence according to the diagnostic methods used. For instance, when the polymerase chain reaction technique was introduced as a method of diagnosis in the same district in 2014, the prevalence of *A. lumbricoides* was reported to be 96% among 662 schoolchildren (see Table 1) [34].

**Fig. 2:**
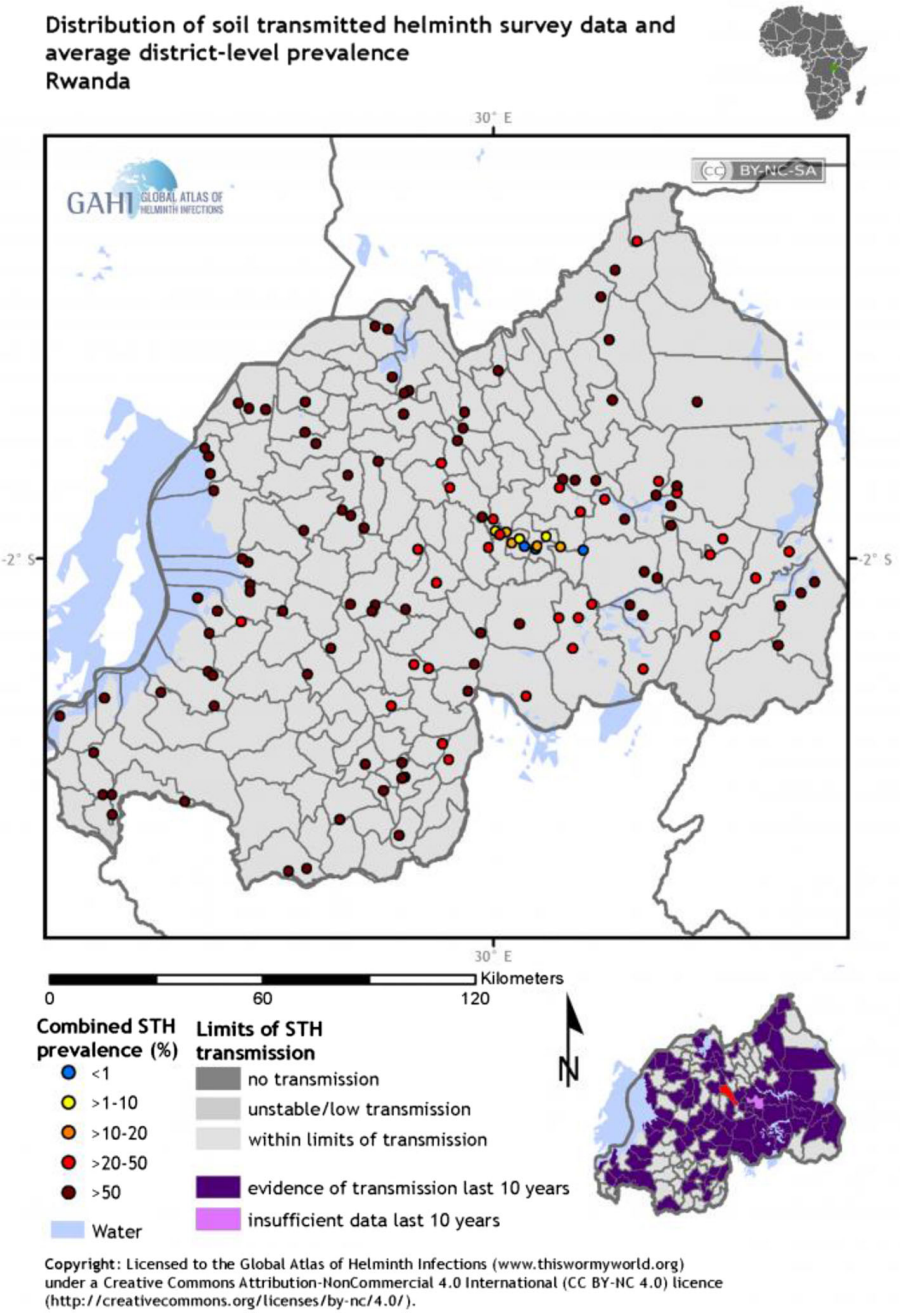
Distribution of STHs in Rwanda. The map shows the maximum point prevalence of STH infections and locations of STH surveys in Rwanda. http://www.thiswormyworld.org/maps/by-worm/soil-transmitted-helminths

In general, the national mapping of 2008 showed a higher prevalence of *A. lumbricoides* and *T. trichiura* in the western and northwestern part of the country, whereas there was a high prevalence of hookworms in the eastern part of the country. The observed variation in distribution may partly be related to ecological differences (the eastern part being warmer and drier than the western part) in the country. Figure 2 shows the distribution pattern of STHs in Rwanda.

The comparison of the geographical distribution of STHs between urban and rural areas indicates that the majority of infections occur in rural areas in 2014 (prevalence of 38% in rural areas versus 13% in urban areas) [34]. The variation in the level of sanitation and hygiene may explain the observed difference in prevalence. The factors associated with the geographical variations in prevalence and the species of STHs in Rwanda have not yet been thoroughly investigated. However, these factors have been well described for other highly endemic countries in Asia and SSA [14, 15].

## Control of intestinal schistosomiasis and STHs

In 2007, through its Ministry of Health, Rwanda initiated a control programme for NTDs with support from other partners such as the SCI, Geneva Global, the Global Network for NTDs, Colombia University and the Access Project [21, 27, 36]. The main objective of the control programme was to reduce the prevalence and morbidities associated with STH infections (ascariasis, trichuriasis and hookworms) and schistosomiasis [36]. Other NTDs targeted were trachoma, lymphatic filariasis and onchocerciasis [21]. To adequately plan a control programme, particularly while using the MDA approach, it became necessary to understand the precise distribution of these infections in the country. Thus, a countrywide mapping exercise was undertaken between 2007 and 2008, whereby the endemic areas were identified [21, 27].

Following the initial 2008 mapping, the first round of MDA was implemented at the national level against STHs in all districts; MDA against intestinal schistosomiasis was only implemented in areas with a prevalence among SAC equal to or higher than 10% [36].

**Fig. 3:**
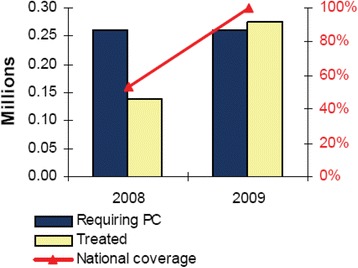
Schistosomiasis treatment coverage for the period 2008–2009 among SAC in Rwanda. Preventive Chemotherapy (PC)- population requiring preventive chemotherapy for schistosomiasis refers to estimates of the number of children needing preventive chemotherapy and the number of treatments for a given period. National coverage refers to the proportion (%) of individuals in the population requiring preventive chemotherapy against schistosomiasis who have been treated

**Fig. 4:**
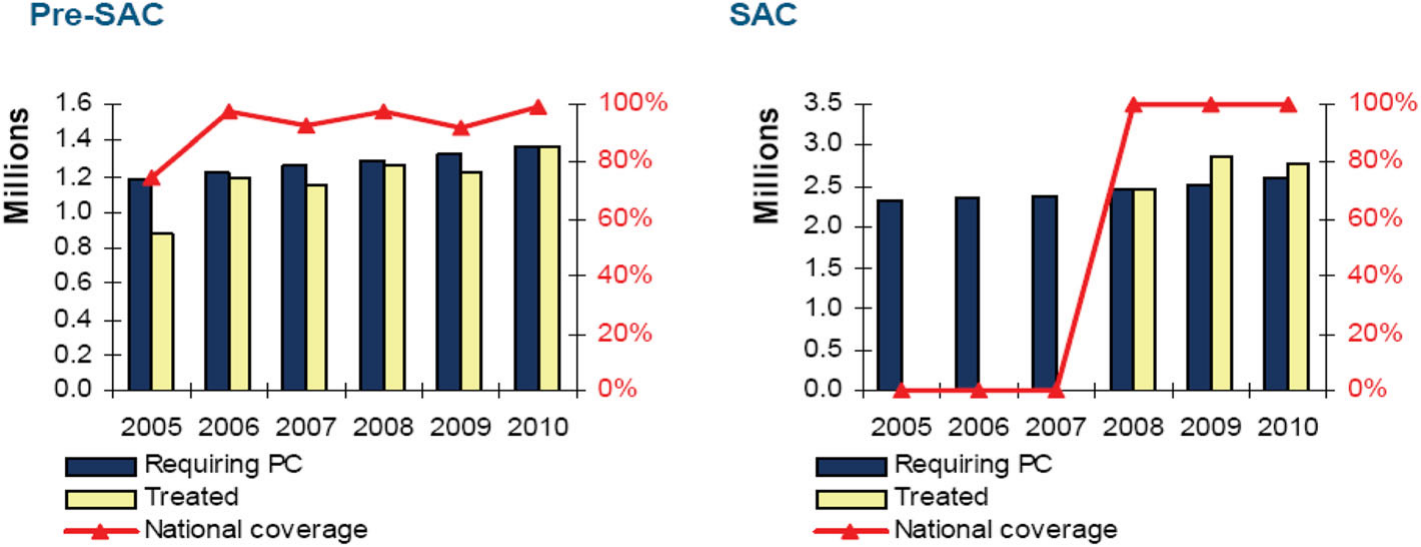
Treatment coverage for STHs for the period 2005–2010 among SAC in Rwanda No data were available on treatment coverage and national coverage for the period of 2005–2007 among SAC. Preventive Chemotherapy (PC)- population requiring preventive chemotherapy for schistosomiasis refers to estimates of the number of children needing preventive chemotherapy and number of treatments for a given period. National coverage refers to the proportion (%) of individuals in the population requiring preventive chemotherapy against schistosomiasis who have been treated

In the first three years of the MDA programme, Rwanda administered over 18 million doses of albendazole/mebendazole (ALB/MEB) against STHs to an estimated 4.2 million individuals; this number corresponding to nearly half of the Rwandese population (in 2010, the estimated total population was 10 277 212) [37]. Figures 3 and 4 show the population that needed treatment and that which was actually treated for the period of 2008–2010 for schistosomiasis and 2005–2010 for STHs. Table 2, created using World Health Organization (WHO) country epidemiological data on NTDs, indicates that for the first round of ALB/MEB, the MDA programme attained a 100% coverage, especially among PreSAC and SAC [37]. In the same round of treatment in 2008, a total of 201 116 SAC and adults received a combination of ALB/MEB and praziquantel (PZQ) and in 2009, 275 650 SAC received a combination of ALB/MEB and PZQ against STHs and schistosomiasis [37]. In 2014, a total of 2 298 684 individuals were estimated to require preventive chemotherapy for schistosomiasis; of these 1 424 532 individuals were SAC. The MDA programme achieved a programme coverage of 98.35% and a national coverage of 7.6% (see Table 3) [38]. For STH infections, with two rounds of MDA with ALB/MEB targeting PreSAC, the programme achieved a 96% programme coverage and a 100% national coverage [38]. Similarly, the MDA programme targeting SAC for two rounds achieved 98.35 and 95.26% programme coverage, and 7.51 and 100% national coverage, for Rounds 1 and 2, respectively (see Table 3) [38].

**Table 2:** Implementation of preventive chemotherapy for the period 2006–2010 using ALB/MEB against STHs among PreSAC and SAC in Rwanda

	2006	2007	2008	2009	2010
	PreSAC	PreSAC	PreSAC/SAC	PreSAC/SAC	PreSAC/SAC
Round 1					
Population targeted	1 105 847	1 127 484	3 895 451	-	-
Population treated	1 193 428	1 185 814	3 580 726	4 088 084	4 129 227
Geographical coverage			100%	-	-
Programme coverage	107.9%	105.2%	91.9%	-	-
Round 2					
Population targeted		1 127 484			
Population treated		1 158 318		2 094 325	
Geographical coverage		-		-	
Programme coverage		102.7%			

○ Geographical coverage - proportion (%) of endemic districts covered with preventive chemotherapy

○ Programme coverage - proportion (%) of individuals who were treated as per the programme target

○ PreSAC- Pre-school Aged Children

○ SAC- School Aged Children

**Table 3:** Implementation of preventive chemotherapy in 2014 using ALB/MEB/PZQ against STHs and schistosomiasis among PreSAC and SAC in Rwanda

Age group	Type of worm infection	Population requiring preventive chemotherapy	Number of children targeted	Reported number treated	Programme coverage	National coverage
Pre-SAC	Round 1- Albendazole/mebendazole					
	STH	948,314	1,295,177	1,252,185	96.68%	100%
		Round 2- Albendazole/mebendazole				
		948,314	1,295,177	1,241,007	95.82%	100%
School aged children	Schistosomiasis/STH	Round-1 Albendazole/Praziquantel				
		2,326,209	177,688	174,762	98.35%	7.51%
		Round-2 Albendazole/mebendazole				
		2,326,209	3,177,065	3,026,425	95.26%	100%

The widespread treatment coverage resulted in high cure rates in the treated children, especially against STH infections [36]. For instance, in the Huye district, the cure rate against STHs was 92% in 2014 and for *A. lumbricoides*, it was 100% [34]. In 2008, administration of one round of PZQ treatment to 2 166 schoolchildren living in endemic districts resulted in a significant decrease in the prevalence of schistosomiasis, from 11.2% at baseline to 2% at one-year follow-up [39]. Similarly, in the same period, the intensity of infection decreased by two folds from 72 eggs per gram of faeces (epg) to 36 epg [39]. Similarly, the prevalence of *A. lumbricoides* infection in Huye district decreased from 61% during the mapping period, to 38% in rural areas and 13% in urban areas following two rounds of treatment [36].

However, despite achieving almost 100% coverage of ALB/MEB and PZQ treatment in 2008, the transmission of STHs and schistosomiasis continues to occur in different epidemiological settings [24, 25, 34, 35]. This not only necessitates repeated treatment, but also the integration of other preventive measures into the MDA programme.

## Discussion

The available data indicate that Rwanda remains a highly endemic area for STH and schistosome infections despite the fact that the country has made significant efforts in mapping and implementing control programmes, especially in the past 10 years. Despite achieving a wide coverage of the MDA programme, the transmission of STHs and schistosomiasis remains a significant public health problem. For instance, the transmission of STHs in Huye district continues to occur despite two rounds of MDA leading to significant cure rates [34]. The Huye district was noted in the mapping programme as being a highly affected area, with a prevalence of *A. lumbricoides* reaching 92% pre-treatment [21, 27]. Similarly, the transmission levels of *S. mansoni* was noted in 2015 as being on the increase on Nkombo Island, in the Rusizi district [24].

The persistent transmission of STHs and schistosomiasis, despite the annual MDA programme, calls for a multisectoral approach for controlling these infections [13]. Depending only on preventive chemotherapy as the main control measure against STHs and schistosomiasis may not reduce the transmission of these infections and their related morbidity. The integration of other public health interventions such as public health education focusing on behavioural changes and the introduction of water, sanitation and hygiene (WASH) measures may enhance the effect of MDA in controlling STHs and schistosomiasis [13].

It has recently been prioritised to implement WASH measures to complement the MDA programmes targeting STH infections in endemic settings [13]. The WASH programme was introduced in Rwanda in 2011 with the help of World Vision in an attempt to tackle water- and hygiene-related diseases. Through the Rwanda WASH programme, a large number of water points have been created or restored, and hand washing and sanitation facilities have been availed to targeted rural communities. However, so far little is known about the impact of WASH efforts on the prevalence and intensity of schistosomiasis and STH infections.

Importantly, designing and implementing an array of public health measures requires an adequate understanding of the local knowledge and perception of the affected population concerning the infections that may affect them [40, 41]. To date, only one study has investigated local knowledge and perception of the communities towards these infections in Rwanda [40]. Comprehending the community level of knowledge and perception is a step towards designing and implementing public health intervention measures [13]. Several methods of public health education have been designed and used in other endemic countries and have resulted in an increased awareness and adherence to control measures [42]. However, it has been reported that public health education provided in the form of posters or teaching aids did not result in changes in knowledge and attitudes towards infections in specific high-risk groups [43].

Between 2000 and 2010, integrated control measures have been implemented in China against schistosomiasis, which included preventive chemotherapy, environmental management, water and sanitation improvement and public health education, have resulted in a significant decrease in the prevalence rate of *S. japonicum* infection to under 1% [44]. The WHO has repeatedly emphasised the importance of integrating a set of public health measures, such as the use of safe water, environmental management, improving sanitation and public health education focusing on behavioural changes, into the MDA programme as part of the control measures against STHs and schistosomiasis [37].

The use of molluscicides (taking their environmental and ecological impact into consideration), which target intermediate host snails that transmit a larval form of *Schistosoma* to the human population, is another approach that can be used on a small scale to reduce or stop the transmission of schistosome infections. However, in Rwanda, information is scarce on the distribution of the intermediate host snails responsible for transmitting *S. mansoni* infection [23], with a few studies implicating the genus *Biomphalaria* [23]. Thus, as the geographical remapping of STHs, schistosomiasis and other NTDs is underway in Rwanda, there is also a need to identify potential intermediate host snails involved in the life cycle of the parasite and to map potential areas for transmission that can be targeted by molluscicides. Studying the physical chemical properties of water bodies with high numbers of snails, as well as aquatic vegetation on which the snails attach, would also be invaluable in predicting high-risk areas. Historical reviews have indicated the importance of molluscicides and suggest that informed snail control can be an effective means of reducing local transmission [45, 46]. In fact, these reviews indicated that widespread snail control using molluscicides has reduced schistosomiasis prevalence in programmes incorporating little or no snail control, respectively [45, 46]. When properly implemented, snail control using molluscicides can complement MDA and reduce rates of re-infection [45, 46].

## Conclusion

Overall, STHs and intestinal schistosomiasis are endemic in Rwanda. While STHs are widely distributed in the country with varying prevalence rates, intestinal schistosomiasis is focally distributed and only endemic along large water bodies. The country has made efforts to understand the distribution of these infections through countrywide geographical mapping using SAC as the main infected group.

The countrywide MDA programme has resulted in a decreased prevalence and intensity of STH and schistosome infections. However, transmission is continuing in most of the endemic districts resulting in re-infection of the treated population. With mathematical models and analysis of the impact of climate change predicting an increasing risk for schistosomiasis transmission in Rwanda in the coming years [19], an integrated approach to control STHs and schistosomiasis is imperative.

The observed high cure rates and reduction in prevalence cannot be sustained only by MDA programmes because these programmes do not modify the environment where transmission occurs. In addition, the current MDA programme only focuses on a segment of the population, that is PreSAC and SAC, and the other segment of the population is not considered, that is, adults who may serve as source of infection to treated children. In addition, in endemic areas, re-infection following treatment occurs very rapidly because of poor sanitation and hygienic practices, and this necessitates re-treatment over a short period of time. Thus, integrating other public health measures into the MDA programme will ensure maintenance of the prevalence of these infections at a low level of transmission after MDA, resulting in a subsequent reduction in re-infection rates.

## Additional file

Additional file 1: Multilingual abstracts abstract in the six official working languages of the United Nations. (PDF 779 kb)

## Abbreviations

ALB: Albendazole
epg: Eggs per gram of faeces
MDA: Mass drug administration
MEB: Mebendazole
NTD: Neglected tropical disease
Pre-SAC: Preschool-aged children
PZQ: Praziquantel
SAC: School-aged children
SCI: Schistosomiasis Control Initiative
SSA: Sub-Saharan Africa
STH: Soil-transmitted helminth
WASH: Water, sanitation and hygiene
WHO: World Health Organization
