# Supplementary material for: Schistosomiasis and soil-transmitted helminthiasis in Rwanda: an update on their epidemiology and control
Source: Infect Dis Poverty. 2017 Mar 1;6:8. doi: 10.1186/s40249-016-0212-z (PMC5331630; doi:10.1186/s40249-016-0212-z)
Supplement: Additional file 1: — Multilingual abstracts abstract in the six official working languages of the United Nations. (PDF 779 kb) [file 40249_2016_212_MOESM1_ESM.pdf]

Translation of the abstract into the six official working languages of the United Nations

داء البلهارسيا والدُّواد المنقول عن طريق التربة في رواندا: تقرير حديث عن مدى تواجدهما والسيطرة عليهما

نادين روجني، دومينيكا مورونا، يوجين روبرانزيزا، همفري مازيغو.

ملخص:
رغم وقوع رواندا في منطقة تتسم بنسبة عالية من داء كل من البلهارسيا والديدان الطفيلية المنقولة عبر التربة، يظل البلاد مفتقرا إلى بيانات وبائية متعلقة بهما. يسعى التقرير التالي إلى جمع البيانات المتاحة عن البلهارسيا وداء الديدان الطفيلية المنقولة عبر التربة في الفترة بين 1940 و2014، وذلك لإعطاء صورة عن واقع تواجد وانتشار هذه العدوى. وسوف تكون هذه المعلومات بدورها داعمة لعملية إعداد وتنفيذ إجراءات مستدامة للسيطرة عليها. تشير السجلات إلى أن البلهارسيا المانسونية والأنواع الرئيسية من الديدان الطفيلية المنقولة عبر التربة هي فقط المتواجدة في رواندا. ذكرت التقارير أنه في عام 2008م كانت نسبة تواجد البلهارسيا المانسونية 2.7%، بمدى يتراوح من صفر إلى 69.5%، أما بالنسبة لداء الدُّواد فكانت 65.8% (التشخيص باستخدام تقنية كاتو-كاتز). تختلف نسبة تواجد هذه العدوات من إقليم إلى آخر، ويظل الأطفال في عمر المدرسة من الفئات الأكثر عرضة لها.
الإجراء الأساسي المستخدم للسيطرة على هذين الداءين هو المعالجة على نطاق واسع بعقاري ألبندزول وبرازيكوانتل، مستهدفين أطفال المدارس بالدرجة الأولى. وفي عام 2008م، شمل البرنامج العلاجي أيضا البالغين الذين يقطنون في المناطق التي يتواجد فيها البلهارسيا المانسونية بنسبة ≥ 30%. ولكن على رغم تمكن رواندا من انجاز المعالجة بنسبة تصل إلى 100% في برنامج العلاج الشامل، فلا يزال انتقال الداءين مستمرا بدلالة نتائج المسوحات الحديثة. ولكي تحصل رواندا على برنامج مستدام للسيطرة على البلهارسيا والديدان الطفيلية المنقولة عبر التربة والقضاء عليهما، فعلى الدولة مراجعة البرنامج المستخدم حاليا واتخاذ برنامجا موحدا يشمل عدة إجراءات متنوعة.

Translated from English version into Arabic by SAlkhodair, through

TRANSLATORS WITHOUT BORDERS

卢旺达血吸虫病和土源性蠕虫病：其流行病学和防治的最新概况

Nadine Rujeni, Domenica Morona, Eugene Ruberanziza, Humphrey D. Mazigo

摘要
尽管卢旺达是血吸虫病和土源性蠕虫病（STH）高度流行区，但该国这些寄生虫病的流行病学资料很少。本综述尝试汇编 1940-2014 年间血吸虫病和 STH 现有数据，以了解这些寄生虫病的流行病学概况。这些信息反过来也将会支持可持续控制措施的设计和实施。现有记录表明，卢旺达仅有曼氏血吸虫（*Schistosoma mansoni*）和蛔虫、钩虫和鞭虫疫情。据报道，2008 年，曼氏血吸虫病的全国患病率为 2.7%（0%-69.5%），使用 Kato-Katz 技术诊断 STH，其总患病率为 65.8%。这些寄生虫病患病率因地而异，学龄儿童仍是高度受影响的群体。主要的控制方法是群体服用阿苯达唑和吡喹酮，主要针对学校中的学龄儿童。2008 年，在曼氏血吸虫感染率≥30%的地区，成年人也被列入群体治疗项目中。然而，尽管卢旺达在 2008-2010 年间几乎实现了该防治方案的全覆盖，曼氏血吸虫和 STHs 的传播仍在继续，最近的调查也证明了这一情况。如果卢旺达要实现血吸虫病和 STH 可持续控制直至消除，需要修订该国的控制策略，采用综合措施，实施综合控制方法。

Translated from English version into Chinese by Jin Chen, edited by Pin Yang

CHINESE CENTER FOR DISEASE CONTROL AND PREVENTION
NATIONAL INSTITUTE OF PARASITIC DISEASES

Bilharziose et les helminthiases transmises par le sol au Rwanda: mise à joursur leur épidémiologie et leur contrôle

Nadine Rujeni, Domenica Morona, Eugene Ruberanziza, Humphrey D. Mazigo

Résumé

Bien que le Rwanda soit situé dans une région qui a une prévalence élevée de bilharziose et d'helminthiases transmises par le sol (STH), les informations épidémiologiques sur ces infections dans le pays restent rares. Cet article vise à compiler les informations disponibles sur la bilharziose et les STH, de 1940 à 2014, afin d'éclairer le profil épidémiologique de ces infections. Cette information pourra ainsi appuyer la conception et la mise en œuvre de mesures de contrôle durables. Les archives actuelles indiquent que seulement *Schistosoma mansoni* et toutes les espèces principales de STH sont endémiques au Rwanda. En 2008, la prévalence nationale de *S. mansoni* a été déclarée être à 2,7%, variant de 0% à 69,5%, et que celle des infections STH était de 65,8% (diagnostic fait par la technique de Kato-Katz). La prévalence de ces infections varie d'un district à l'autre, mais les enfants d'âge scolaire sont toujours un groupe très largement affecté. La principale méthode de contrôle est l'administration massive de médicaments (albendazole et praziquantel), ciblant principalement les enfants d'âge scolaire au niveau des écoles. En 2008, les individus adultes vivant dans des zones avec une prévalence de *S. mansoni*⩾30% ont aussi été inclus dans le programme d'administration massive de médicaments. Cependant, bien que le Rwanda atteigne une couverture de presque 100% de son programme en 2008-2010, la transmission de *S. mansoni* et des STH continue d'avoir lieu, ce qui illustré par les plus récentes études. Pour que le Rwanda parvienne à contrôler et éliminer durablement la bilharziose et les STH, il est nécessaire de revoir la stratégie de contrôle du pays et d'adopter une approche de contrôle intégré, qui implique une combinaison de mesures.

Translated from English version into French by AdrienneDaudet, through

Шистосомоз и гельминтозы, передаваемые через почву, в Руанде: обновленная информация по эпидемиологии и контролю

Надин Руени, Доменика Морона, Юджин Руберанзиза, Хамфри Д. Мазиго

Краткий обзор

Несмотря на то, что Руанда находится в регионе с высокой распространенностью шистосомоза и гельминтозы, передаваемой через почву (STH), эпидемиологической информации об этих инфекциях в стране мало. Настоящий обзор ставит перед собой задачу сбора имеющихся данных о шистосомозе и STH с 1940 по 2014 год, и предоставления обзора, способного дать представление о эпидемиологических сведениях этих инфекций. Эта информация будет, в свою очередь, поддерживать разработку и реализацию контрольных мер. Доступная информация показывает, что только *Schistosoma mansoni* (Шистосома Мансона) и все основные виды STH являются эндемичными в Руанде. В 2008 году национальный показатель распространенности *S. mansoni*, как сообщается, был 2,7%, в диапазоне от 0% до 69,5%, и что инфекции STH составили 65,8% (диагноз с помощью метода Като-Кац). Распространенность этих инфекций варьируется от района к району, наиболее подверженной группой являются школьники. Основной метод контроля – массовое применение препаратов Альбендазол и Празиквантел, в основном распространяемые среди детей школьного возраста и в школьной среде. В 2008 году более 30% взрослых людей, живущих в районах с преобладанием *S. mansoni*, были также включены в программу массового применения препаратов. Не смотря на то, что Руанда достигла практически 100% покрытия этой программы в 2008-2010 гг., передача *S. mansoni* и STH продолжает иметь место, что отображено в последних опросах. Если Руанда планирует достигнуть устойчивого контроля и устранения шистосомоза и STH, существует необходимость пересмотра стратегии государственного контроля и принятия комплексного подхода к контролю, который предусматривает сочетание мер.

Translated from English version into Russian by Semen Abramov, through

Esquistosomiasis y helmintiasis transmitido por el suelo en Ruanda: Una actualización sobre su epidemiología y su control

Nadine Rujeni, Domenica Morona, Eugene Ruberanziza, Humphrey D. Mazigo

Resumen

Aunque Ruanda se encuentra dentro de una región de alta prevalencia de esquistosomiasis y de infecciones de helmintos transmitidos por el suelo (HTS, o STH en inglés), la información epidemiológica sobre estas infecciones en el país es poca. El actual análisis intenta recopilar las estadísticas disponibles sobre el esquistosomiasis y los HTS, de 1940 a 2014, para proveer un conocimiento sobre el perfil epidemiológico de estas infecciones. Esta información entonces apoyará el diseño y la implementación de métodos de control sostenibles. Los registros disponibles indican que sólo el *Schistosoma mansoni* y todas las especies mayores de los HTS son endémicos de Ruanda. En 2008, la prevalencia nacional de *S. mansoni* se informó al 2.7%, que va desde el 0% al 69.5%, y que el de los HTS era el 65.8% (diagnosticado usando la técnica Kato-Katz). La prevalencia de estas infecciones varía de región en región, con los niños de edad escolar permaneciendo el grupo más afectado. El método principal de control es la administración en masa de fármacos usando albendazole y praziquantel, enfocándose mayormente en los niños de edad escolar en ambientes escolares. En 2008, los adultos que vivían en áreas con una prevalencia de *S. mansoni* $\geqslant$ del 30% también se incluyeron en el programa de la administración en masa de fármacos. Sin embargo, a pesar de que Ruanda logró un alcance del 100% de este programa de 2008 a 2010, la transmisión de *S. mansoni* y los HTS sigue sucediendo, como se ilustró en los estudios más recientes. Si Ruanda quiere lograr el control sostenible y la eliminación de esquistosomiasis y los HTS, se necesita revisar la estrategia de control del país y asumir un método de control integrado que involucre una combinación de medidas.

Translated from English version into Spanish by Kate Pattison, through

TRANSLATORS
WITHOUT BORDERS
